# A Proposed Scalable Design and Simulation of Wireless Sensor Network-Based Long-Distance Water Pipeline Leakage Monitoring System

**DOI:** 10.3390/s140203557

**Published:** 2014-02-20

**Authors:** Abdulaziz S. Almazyad, Yasser M. Seddiq, Ahmed M. Alotaibi, Ahmed Y. Al-Nasheri, Mohammed S. BenSaleh, Abdulfattah M. Obeid, Syed Manzoor Qasim

**Affiliations:** 1 Department of Computer Engineering, King Saud University, Riyadh 11421, Saudi Arabia; E-Mails: mazyad@ksu.edu.sa (A.S.A.); yseddiq@kacst.edu.sa (Y.M.S.); alzpen@hotmail.com (A.M.A.); al_nasheri@yahoo.com (A.Y.A.); 2 National Center for Electronics, Communications and Photonics Research, King Abdulaziz City for Science and Technology (KACST), Riyadh 11442, Saudi Arabia; E-Mails: mbensaleh@kacst.edu.sa (M.B.S.); obeid@kacst.edu.sa (A.M.O.)

**Keywords:** energy-efficient, leakage detection, pipeline monitoring, RFID, simulation, wakeup techniques, wireless sensor network (WSN)

## Abstract

Anomalies such as leakage and bursts in water pipelines have severe consequences for the environment and the economy. To ensure the reliability of water pipelines, they must be monitored effectively. Wireless Sensor Networks (WSNs) have emerged as an effective technology for monitoring critical infrastructure such as water, oil and gas pipelines. In this paper, we present a scalable design and simulation of a water pipeline leakage monitoring system using Radio Frequency IDentification (RFID) and WSN technology. The proposed design targets long-distance aboveground water pipelines that have special considerations for maintenance, energy consumption and cost. The design is based on deploying a group of mobile wireless sensor nodes inside the pipeline and allowing them to work cooperatively according to a prescheduled order. Under this mechanism, only one node is active at a time, while the other nodes are sleeping. The node whose turn is next wakes up according to one of three wakeup techniques: location-based, time-based and interrupt-driven. In this paper, mathematical models are derived for each technique to estimate the corresponding energy consumption and memory size requirements. The proposed equations are analyzed and the results are validated using simulation.

## 1. Introduction

The worldwide losses of water due to the distribution network leakage is estimated at 48.6 billion m^3^, thus causing a monetary loss of approximately 14.6 billion US dollars per year, according to a World Bank study [1]. Water is an important and limited resource, hence it is necessary to protect and use the water utilities efficiently. Water leakage is considered to be one of the largest and most serious challenges. It is expected to intensify over time, given the scarcity of available traditional water resources and the enormous costs of providing fresh potable water from non-traditional sources such as desalination plants. Long-distance water pipelines have become an indispensable part of such infrastructure. Active monitoring and inspection is required to maintain the health of the pipelines [2,3]. A pipeline monitoring system has a long list of tasks to accomplish. In addition to detecting and localizing leakage and bursts, these tasks include measuring pipe cross-section and wall thickness and monitoring fluid purity and flow speed [4,5].

Pipeline monitoring tasks become more challenging when applied to long-distance water pipelines covering thousands of kilometers. Several issues should be considered, such as the difficulty of the maintenance of the static components and efficiency of memory usage and energy consumption. Wireless sensor networks (WSNs) provide an efficient way to address these issues. To the best of our knowledge, the problem of monitoring long distance water pipelines using WSNs has not been properly addressed in the published literature, despite its requirement in the practical field.

Different type of sensors, such as temperature sensor, pressure sensor, acoustic sensor, flow sensor, and pH sensor are typically used for water pipeline monitoring. These sensors generate appropriate electrical signals based on the sensed phenomena. Generally, monitored parameters include temperature, humidity, flow and pressure. Selecting an appropriate sensor or sensing technique depends on many aspects such as the pipeline material and environment (aboveground or underground).

A typical WSN node consists of a sensing subsystem, a processing subsystem, a communication subsystem and a power supply subsystem. The processing subsystem which mainly comprises of microcontroller and memory processes and stores the sensor data respectively. The RF transceiver, which is an important part of communication subsystem receives commands from a central computer and transmits the pr maintenance of the static components ocessed data to that computer. The power for each WSN node is derived from a battery or an energy harvesting (scavenging) device.

In this paper, a scalable design and simulation of a long-distance above ground water pipeline leakage monitoring system using WSN is proposed. The challenges of difficult maintenance, efficient memory usage and low energy consumption are considered. The design is based on deploying mobile sensor nodes that are driven by the water current. A multi-node model is adopted in order to make the design scalable for various distances, memory sizes and battery lifetimes. Among the deployed nodes, one should be in-duty while the others are sleeping. At a certain stage, the active node turns itself off after it hands over the task to another node. Task handover takes place using the following three methods: location-based, time-based and interrupt-driven. Localization is done using Radio Frequency IDentification (RFID) tags that are placed at fixed positions outside the pipeline. Mathematical models are also derived to estimate the energy consumption and the memory usage of the proposed design.

The rest of the paper is organized as follows: Section 2 presents the related work. The proposed design is described in Section 3. The mathematical models are discussed and analyzed in Section 4. Section 5 presents the simulation results of the proposed system. The results are validated through Matlab simulation in Section 6. Finally, conclusions and recommendations for future work are discussed in Section 7.

## 2. Related Work

A number of WSN-based solutions for pipeline monitoring have been proposed in the literature [2,3]. Jawhar *et al.* presented an initial framework for using linear WSNs for oil, gas, and water pipeline monitoring applications [6]. PipeNet is one such system which is used for automated detection, localization and quantification of leaks, bursts and other anomalies in large diameter bulk water transmission pipelines [7]. Accelerometer sensors are used to measure the vibrations that can result from the presence of cracks in the pipeline. PipeNet provides near real-time operation with few false alarms. A scalable, non-intrusive, autonomous and adaptive water monitoring system (NAWMS) is presented in [8]. It detects and locates leakages using low cost wireless vibration sensors that are externally attached to the pipes. It can be used to estimate the water consumption with minimum error.

An autonomous pipeline monitoring system called TriopusNet is presented in [9]. Sensor nodes are automatically released from a centralized repository located at the source of the water pipeline and carried forward by the water flow. The nodes are placed automatically based on a sensor deployment algorithm. Each sensor node includes a motor which allows the three arms to latch onto the pipe's inner surface. This is explained in detail in [9]. Human effort is not required to install and repair sensor nodes in this system.

A fault-tolerant and reliable architecture based on an integrated wired and wireless sensor network for monitoring aboveground pipeline infrastructures is presented in [10]. SPAMMS is a low-cost, scalable, customizable and autonomous sensor-based system which is presented in [11]. This system combines sensing technology with robot agent-based technology to provide active and corrective monitoring and maintenance of the pipelines. SPAMMS combines RFID systems with mobile sensors and autonomous robots to monitor pipelines. Different pipeline monitoring techniques are compared and discussed in this paper [11].

Underground pipelines are mostly preferred to transport water from remote locations. This provides the safest way to transport water, but at the cost of extreme environmental conditions under the ground which may cause leakage on the pipelines [12–14]. A low-cost magnetic induction waveguide-based WSN technique for underground pipeline monitoring (MISE-PIPE) is presented in [12]. In MISE-PIPE, two type of sensors are used, one placed inside and the other placed outside the pipeline. Both internal and external sensors provide sufficient data for detecting and localizing the leakage in the pipeline. The authors claim that this technique can provide accurate real-time leakage detection and improved lifetime for the underground pipelines.

PipeTECT, an intelligent and scalable WSN system for real-time nondestructive monitoring of underground water pipelines is discussed in [15]. MEMS accelerometers on the pipe surface are employed to measure vibrations in order to determine the change in the water pressure caused by pipe rupture and thus localize the leakage. However, it faces some challenges such as reliable long-range communication, precise time synchronization, power management and effective bandwidth usage [15]. The PipeTECT system was further improved by adding new modules at the sensing and data aggregation unit which reduced the total energy consumption significantly [16].

## 3. Proposed Design

This work proposes a non-real-time leakage monitoring system for long-distance water pipelines. A mobile sensor node is allowed to move with the water current from the pipeline source down to the sink where the node is collected and its memory content is copied to a computer. This data contains all the sensor and location readings that are taken by the node throughout its long trip inside the pipeline. The node observation is subjected to offline analysis to locate the leakage.

A node records its location based on its exposure to signals of RFID tags that are placed in fixed position outside the pipeline surface. The number of tags used is inversely proportional to the distance between tags (Δ*d*). If the total pipeline distance is *D*, then the number of RFID tags required for the whole system (*M*) can be calculated as follows:

(1) $$M=\left[\frac{D}{\Delta d}\right]$$

The use of active RFID tags, which are battery-operated, enables their signal to penetrate through the pipeline walls. Some active RFID tags today are able to transmit up to 200 m in free space (e.g., the ZT-50) [17]. Thus, tag signal penetration through few tens of centimeters of the pipe wall would be possible provided that the pipeline material allows signal propagation. Nevertheless, this assumption is not valid for strongly isolating materials such as steel.

**Figure 1.**
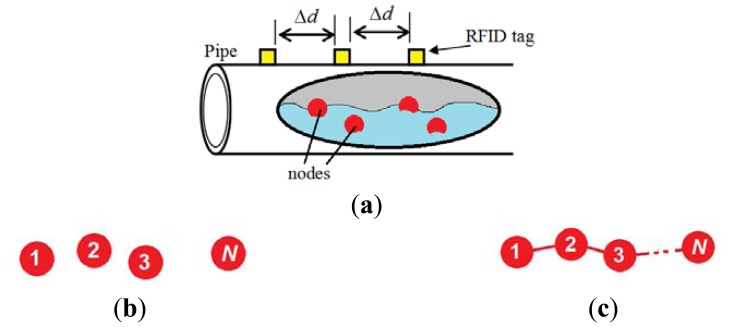
(**a**) Proposed design components; (**b**) Loose independent nodes; (**c**) Nodes connected in series using wires (For interrupt-driven method).

Basically, active RFID tags are battery-operated, which implies the need for replacing their batteries from time to time. That would not be easy when considering long-distance pipelines that pass through rural and difficult to approach areas. For that reason, solar cells can be used as a renewable power source for the active RFID tags. A general illustration of the proposed design is shown in Figure 1.

In order to add scalability and efficiency to the system and to simplify the node design, a multi-node model is adopted in this design. That is, during the trip period (*T*) in the pipeline, a group of *N* nodes, where *N* > 1, are deployed and allowed to move with the water current. These nodes work cooperatively to perform the monitoring tasks by allowing only one node to be in duty for a certain interval *T_A_* while the other nodes are inactive. The active node gets busy sensing and localizing leakages before it cuts off after a period of *T_A_* since it commenced its mission. That active period (*T_A_*) is determined as follows:

(2) $$T_{A}=\frac{T}{N}$$

The inactive nodes are either totally off, if they already finished duty, or sleeping, if they are awaiting their turn to start duty. Duty handover from one node to another takes place using one of the following three methods: location-based, time-based and interrupt-driven.

In the location-based method, the sleeping nodes keep locating themselves while sleeping. Each node knows where it should commence duty and hence wakes up. In contrast, in the second method, which is time-based, the sleeping nodes have to keep their timer on during the sleep period. When the appropriate time of commencing duty comes, the node wakes up. Since, nodes float independent of each other, racing between nodes may occur and it is possible that a node that is about to wake up is way ahead of the node that is currently in duty. In this situation, there will be a pipeline segment, which might have leakage and not monitored by either of the two nodes. A possible solution could be a time overlap between them to reduce the chance of having that problem. Certainly, this redundancy will increase the energy consumption. A more detailed analytical proof of pipeline coverage by the three techniques is provided in the Appendix section.

The third method involves an interrupt-driven wakeup. When using this method, sleeping nodes neither locate nor do they count time. Rather, a sleeping node waits for an interrupt signal from the active node via a wire connecting them. Therefore, when using the interrupt-driven wakeup method, the nodes must be connected in series using wires in a chain as shown in Figure 1c. The series connection may cause a reliability problem. That is, if any node breaks down, all the subsequent nodes will be out of service. Perhaps the use of a wire can be avoided by reusing nodes and having them go into sleep mode while waiting for an interrupt signal. With proper packaging and mechanical design, the node can be made floatable [18].

**Figure 2.**
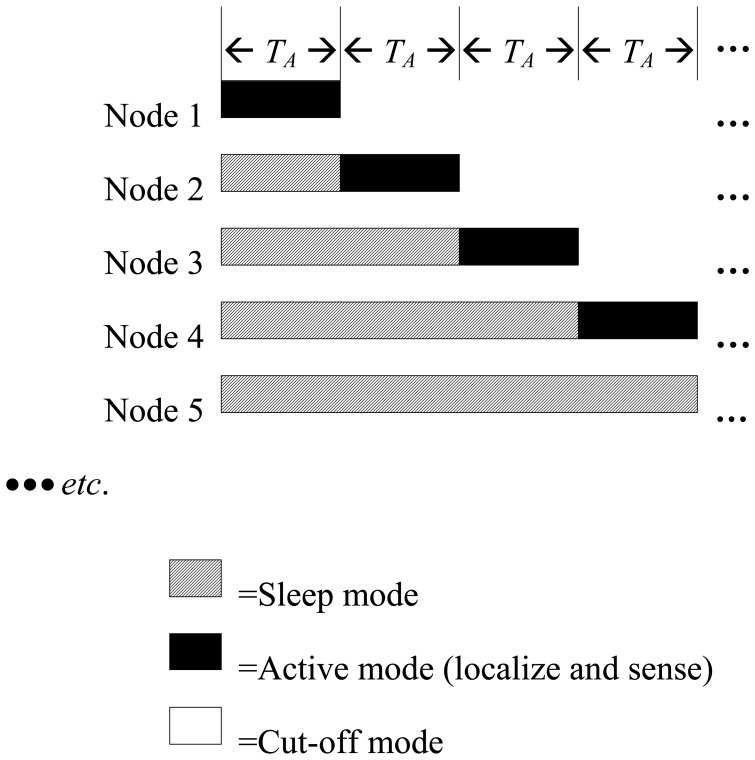
Sleep-active modes of the proposed design.

Regardless of the duty handover method that is used, when an in-duty node finishes its task, it cuts off and it does not perform any activity until it reaches the pipeline sink. The transition from sleep to active modes and from active to cut-off mode is illustrated in Figure 2. Table 1 summarizes the activities during the sleep and active modes.

**Figure 3.**
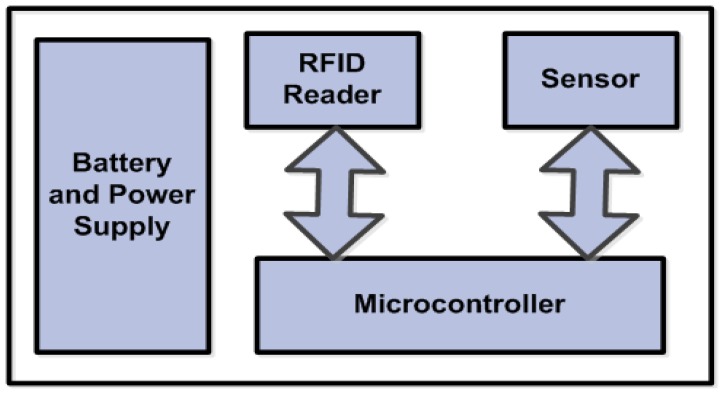
General block diagram of sensor node.

Each node should be equipped with components that enable it to perform its job efficiently. For localization, a node uses an RFID tag reader that can acquire the IDs of the active tags every time the node passes under an active tag. Since, the main purpose of deploying the node is to sense, the presence of a sensor (e.g., pressure or velocity) is necessary. The RFID reader and the sensor are controlled by a low-energy microcontroller. A general block diagram of the sensor node is shown in Figure 3.

## 4. Mathematical Modeling and Analysis

There are many parameters that affect the energy consumption and the memory usage of each node of the aforementioned design. The key parameters are described in Table 2. For energy consumption, the following relationship is used:

(3) E=P×T

where, *E* is the energy consumed during the time period *T* by a system that consumes power *P*.

When deploying *N* nodes in the pipeline, the energy consumption of the *n*th node, where 1≤*n*≤*N*, can be estimated as discussed in the following sections.

### 4.1. Location-Based Wakeup Method

To analyze the total energy consumption of the *n*^th^ node, the energy consumed during the sleeping mode *E*_*n*(*sleep*)_ and active mode *E*_*n*(*active*)_ should be taken into consideration, i.e.:

(4) $$E_{n}=E_{n(\text{sleep})}+E_{n(\text{active})}$$

In the location-based wakeup method, a sleeping node consumes energy in localizing itself which implies that both the RFID reader and the microcontroller are doing some activities. The *n*^th^ node spends a period of (*n* − 1)*T_A_* sleeping. Therefore:

(5) $$E_{n(\text{sleep})}=(n-1)\left(T_{A}\left(P_{rd(\text{idle})}+P_{C}\right)+mT_{rd(A)}P_{rd(A)}\right)$$

The parameters used in Equation (5) and the subsequent equations are described in Table 2. The term *mT_rd(A)_P_rd(A)_* in Equation (5) refers to the energy consumed by the RFID tag reader when communicating with one of the *m* tags that the node will be exposed to during a period of *T_A_*. The relationship between *m* and the total number of the tags (*M*) is given by Equation (6):

(6) $$m=\left[\frac{M}{N}\right]$$

For the active mode of the location-based method, the energy is consumed by all of the node components, i.e., the sensor, the tag reader and the microcontroller. Knowing that the node spends a period of *T_A_* in active mode, the following equation can be deduced:

(7) $$E_{n(\text{active})}=T_{A}\left(P_{rd(\text{idle})}+P_{s}+P_{C}\right)+mT_{rd(A)}P_{rd(A)}$$

Substituting Equations (5) and (7) in Equation (4) will result in the following Equation (8):

(8) $$E_{n}=T_{A}\left(nP_{rd(\text{idle})}+P_{s}+nP_{C}\right)+nmT_{rd(A)}P_{rd(A)}$$

### 4.2. Time-Based Wakeup Method

For the time-based wakeup method, the total energy consumed by the *n*th node can be derived by, first, calculating the sleep mode energy as just the energy consumed by the microcontroller (assuming the timer is implemented as a piece of code). Recalling that the *n*^th^ node spends a period of (*n* − 1)*T_A_* in sleeping mode, the sleep mode energy can be calculated as:

(9) $$E_{n(\text{sleep})}=(n-1)T_{A}P_{C}$$

The active mode of the time-based wakeup method is the same as the location-based wakeup method, i.e., it can be calculated using Equation (7). Thus, Equation (10) can be formed by substituting Equations (7) and (9) in Equation (4):

(10) $$E_{n}=T_{A}\left(P_{s}+P_{rd(\text{idle})}+nP_{C}\right)+mT_{rd(A)}P_{rd(A)}$$

### 4.3. Interrupt-Driven Wakeup Method

Finally, in the interrupt-driven method, a sleeping node does not do any activity while it is in sleep mode. Therefore:

(11) $$E_{n(\text{sleep})}=0$$

The active mode energy under the interrupt-driven method is the same as the other two methods and can be determined using Equation (7). Substituting Equations (7) and (11) in Equation (4) will lead to Equation (12):

(12) $$E_{n}=T_{A}\left(P_{s}+P_{rd(\text{idle})}+P_{C}\right)+mT_{rd(A)}P_{rd(A)}$$

To analyze Equations (8), (10) and (12), several values for the equations variables are assigned according to Table 3. In addition, Table 4 lists the values of the power consumption of the components that can be used to build the sensor node.

To derive a mathematical model for calculating the *n*th node memory size, it should be realized that the only two components that write data to memory are the RFID tag reader and the sensor. Moreover, memory is only written to during the active mode, which lasts for a period of *T_A_*. During that period, the tag reader will communicate to *m* tags and store their IDs to the memory. If an ID consists of *W_RFID_* bytes, then the total number of bytes that are stored in the memory and belong to the tag reader is as follows:

(13) $$S_{n(\text{reader})}=mW_{\text{RFID}}$$

The sensor is the other component that stores data in memory. During the active period (*T_A_*), the sensor performs sensing *f_s_* times per second. Each time, a sample of width *W_sensor_* is stored into the memory. The total number of samples during an active period is *f_s_T_A_* samples. And the total number of bytes that the sensor will store into memory is as follows:

(14) $$S_{n(\text{sensor})}=f_{s}T_{A}W_{\text{sensor}}$$

When adding Equations (13) and (14), the expression of Equation (15) will be formed.

(15) $$S_{n}=mW_{\text{RFID}}+f_{s}T_{A}W_{\text{sensor}}$$

To analyze Equation (15), different values of *N* are assigned as assumed in Table 3. It is also assumed that the sensor node performs sensing twice a second, i.e., *f_s_* = 2 samples per second. Every time it samples, the sensor sample size (*W_sensor_*) is assumed to be two bytes long. For the RFID tag, the tag ID is assumed to consist of 16 bytes. The value of the number of RFID tags per pipeline segment (*m*) can be calculated using Equations (1)–(3), (6), (8), (10), (12).

## 5. Results Discussion

**Figure 4.**
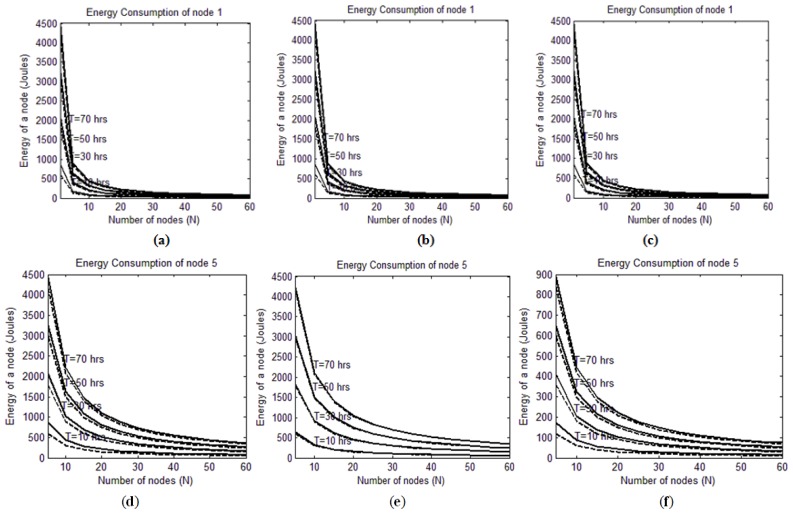

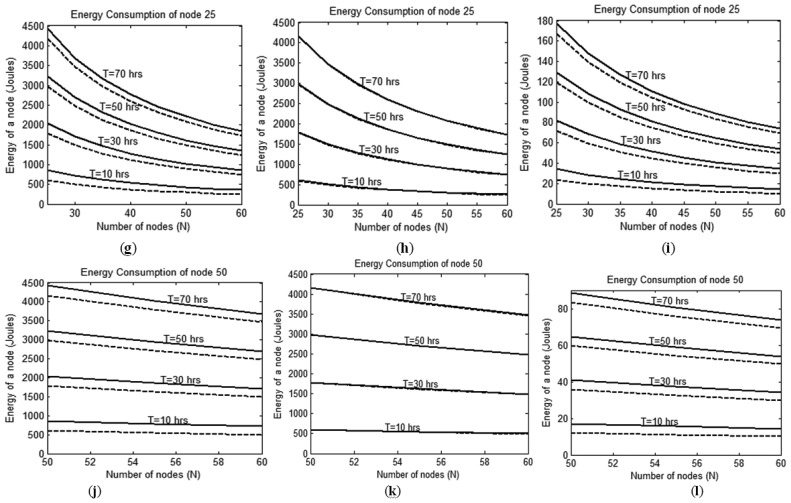
Energy consumption analysis results for (**a**) Location-based wakeup (1st node); (**b**) Time-based wakeup (1st node); (**c**) Interrupt-driven wakeup (1st node); (**d**) Location-based wakeup (5th node); (**e**) Time-based wakeup (5th node); (**f**) Interrupt-driven wakeup (5th node); (**g**) Location-based wakeup (25th node); (**h**) Time-based wakeup (25th node); (**i**) Interrupt-driven wakeup (25th node); (**j**) Location-based wakeup (50th node); (**k**) Time-based wakeup (50th node); (**l**) Interrupt-driven wakeup (50th node).

The analysis results for energy consumption are plotted in Figure 4. The x-axis represents the different values of *N*, which is the number of member nodes in a group, e.g., when *n* = 15, that refers to a 15-node group deployed in the pipeline. The y-axis refers to the energy consumed by a single node that is a member of an *N*-node group. On each plot, there are two types of curves: solid line and dashed line curves that correspond to the minimum and the maximum distances of separation between the RFID tags respectively. That is, the solid line is associated with Δ*d* = 10 m while the dashed line is associated with Δ*d* = 500 m. Moreover, each plot contains four curves that represent total trip times of 10, 30, 50 and 70 h. Figure 4 also consists of twelve plots, from (*a*) to (*l*), arranged in a matrix of four rows and three columns. The first, second and third columns of the matrix depicts the results of analyzing the location-based, the time-based and the interrupt-driven wakeup methods respectively. In other words, the first, the second and the third columns of the matrix depicts the results of analyzing Equations (8), (10) and (12) respectively. Each row of the matrix focuses on analyzing a specific node within the group using the three wakeup methods. That is, the first, second, third and fourth rows refer to the 1st, the 5th, the 25th and the 50th nodes of the group being analyzed respectively.

It can be seen from Figures 4a–c, that the energy consumption of the first node is only affected by the number of nodes in the group and the distance between RFID tags, while the wakeup method has totally no effect on it. That is because the first node is not subjected to the sleep mode as it starts in active mode by default. The three wakeup methods differ only in what a node does before coming to duty and according to the way it wakes up. Obviously, none of these two differences are applicable to the first node.

On the other hand, the plots in the second, the third and the fourth rows of Figure 4 show clearly that nodes energy consumption is dependent on the wakeup method that is used. Consider two sets of plots: the set of plots of Figure 4d, g and j and the set of plots of Figure 4e, h and k. The energy consumption that is depicted in those sets is almost identical. That is because these two sets represent the location-based and the time-based wakeup methods that are both involved in some activity during the sleeping mode. That is, if a node follows the location-based method, it consumes some energy while sleeping to localize itself. Likewise, with the time-based method, some energy is consumed in sleep mode by the node timer. On the other hand, since the interrupt-driven method involves no activity when a node is sleeping, the plots of Figure 4f, i and l are different than the other two methods. As expected, increasing the number of nodes per group will result in a significant drop in energy consumption. Obviously, that is because of the deep sleep mode that characterizes the interrupt-driven methods. Such significant energy saving can overshadow the reliability problem that is associated with the interrupt driven method that was discussed earlier in Section 3.

It is also clear that the difference between a solid line curve and its dashed line counterpart is small. The reason why the energy consumption in the dashed line curves is always less is because the RFID tags are so far from each other (Δ*d* = 500 m). Consequently, the RFID reader does not need to communicate with the tags so often as in the case of solid line curves when Δ*d* = 10 m. However, by increasing Δ*d*, the energy consumption just slightly improves at the expense of significant degradation in localization resolution. Clearly, the insignificant energy saving is not worth that sacrifice in distance resolution.

In general, the energy consumption drops in all the methods, but when a node is scheduled to wake up late (a high value of *n*), that drop becomes less sharp. However, that is not true with the interrupt-driven method, which maintains the significant reduction even with nodes that wake up too late. The justification for this is that when considering the time-based or the location-based methods, a sleeping node still consumes energy to count time and locate itself, which are marginal activities that are not within the core duties of the node.

The longer the sleep period is, the more those marginal activities are performed. In contrast, a node that works under the interrupt-driven method does not suffer from those marginal activities since it is in deep sleep mode and doing absolutely nothing when awaiting its turn to wakeup. The existence of the marginal activities in the location-based and the time-based methods introduces limitations to the design scalability when choosing these two methods. However, the interrupt-driven method is scalable to any number of nodes and any trip period.

**Figure 5.**
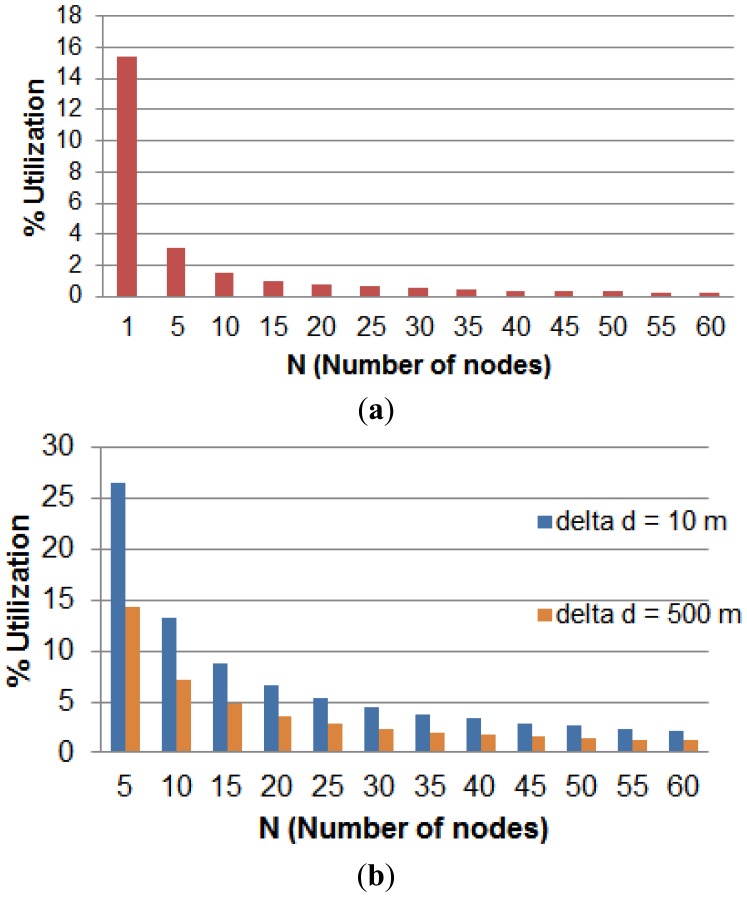
(**a**) Node memory utilization (*T* = 30 h, Δ*d* = 10 m); (**b**) Node memory utilization (When *N* > 1) for *T* = 50 h.

The second part of this analysis is concerned about the nodes memory size. The relationship between memory size and the other parameters is governed by Equation (15). The analysis results are depicted in Figure 5. The x-axis refers to total number of nodes per group while the y-axis refers to the percentage memory utilization for each node. Figure 5a shows that the memory size of a node drops sharply after adopting the multi-node model. The chart of Figure 5b shows the effect of increasing the separation between RFID tags on the node memory usage. When the value of Δ*d* is increased from 10 m to 500 m, the memory size is dropped to almost 50%. This reduction is significant and in case of running short of memory, sacrificing the localization resolution is worth the gain in memory saving.

## 6. Model Validation Using Simulation

An event-driven simulation was carried out using Matlab to validate the analytical models formulated in Equations (8), (10) and (12) and the results presented in Figure 4. The design parameters were tuned according to Table 3. For each combination of parameter values, the simulated energy results (*E_sim_*) were compared with the corresponding analytical results obtained from the proposed equations (*E_eq_*).

The simulation is triggered by some events based on whether the node is in active or sleep mode. While in active mode, the simulation is triggered by an event when the node is exposed to an RFID tag. After every event, the energy is calculated for each component contributing in reading the RFID communication, namely, the microcontroller and the RFID reader. Also, the energy of the pressure sensor is calculated considering operation time throughout the active mode. While in sleep mode, the simulation is triggered by RFID tag exposure as well. However, the energy calculation differs based on the three techniques listed in Table 1. For this scenario, both the simulated and the analytical energy results were compared and found to be identical with zero error.

The simulation was repeated with less event resolution. That is, instead of triggering the simulation by every RFID tag exposure, the simulation is triggered every *T_A_* seconds, where *T_A_* is a node active period calculated using Equation (2). During the time period (*T_A_*), a node is exposed to *m* RFID tags as described in Table 2. Under this scenario, the results were found to be almost identical, with a very small error. The error was calculated by taking the absolute value of the difference of the simulated and analytical results, and as the percentage of the absolute error relative to the simulation results. That is:

(16) $$\text{AbsoluteError}=\left|E_{\text{sim}}-E_{eq}\right|$$

(17) $$\text{RelativeError}=\frac{\left|E_{\text{sim}}-E_{eq}\right|}{E_{\text{sim}}}\times 100\%$$

**Figure 6.**
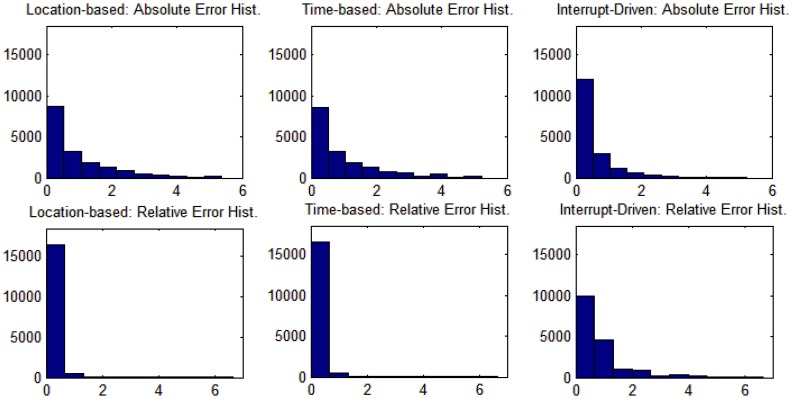
Histogram results of the error analysis.

Table 5 summarizes the comparison results of the whole experiment using the three wakeup techniques. The histogram results of the error analysis are also shown in Figure 6.

## 7. Conclusions

Water pipeline networks are considered to be an important asset which requires active monitoring and inspection systems for maintenance. Monitoring long distance water pipeline for leakages, bursts and other anomalies is a challenging task which requires energy-efficient, scalable and robust mechanisms. WSN is one such technology which provides robust solutions to these problems. In this paper, a scalable design and simulation of a non-real-time RFID-WSN-based long-distance aboveground water pipeline leakage monitoring system is presented. The system is based on deploying multiple mobile sensor nodes such that only one node is active for specific period of time. While a node is active, the other nodes are in sleep mode. Sleeping nodes wakeup using three different techniques: location-based, time-based and interrupt-driven. After finishing duty, a node cuts off until the end of its trip. Mathematical models for the energy consumption and the memory usage are proposed, analyzed and validated using Matlab simulation. The analysis results shows that energy consumption of the node improve significantly when the work is divided between groups of nodes. Although the energy consumption can be further improved by increasing the distance between RFID tags, that improvement is not significant and it is overweighed by the significant degradation in distance resolution.

The energy savings that are associated with the location-based and time-based wakeup methods are almost the same. On the other hand, the interrupt-driven method provides a much sharper reduction in energy consumption. This advantage may overshadow the reliability issue of this method due to the fact the nodes are connected in series. One more advantage of the interrupt-driven method is its scalability, even if the number of nodes is high and the trip period is long. In contrast, the other two wakeup methods have limited scalability due to the marginal activities that node need to perform in their sleep mode.

Memory size is also improved when adopting the multi-node model. Unlike the energy saving case, memory saving is also significant when increasing the distance between RFID tags. This analytical work is the first step towards a long term project with objectives of developing an efficient and fault-tolerant monitoring system for a long-distance water pipeline. As a future work, more investigation and development will be carried out to prototype an energy efficient WSN node for water pipeline leakage monitoring system. An experimental testbed based on the resulting node will also be developed and tested using real-world scenarios.

## Figures and Tables

**Figure A1.**
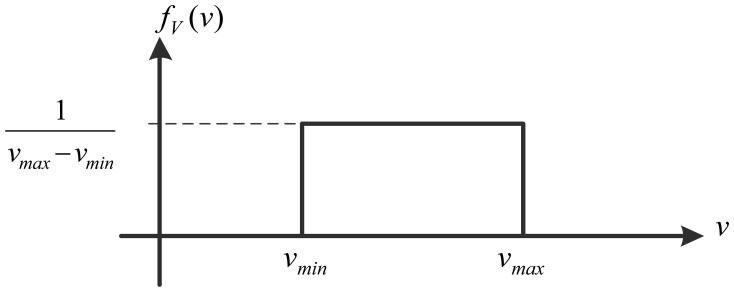
The density function of the random variable *v*.

**Figure A2.**
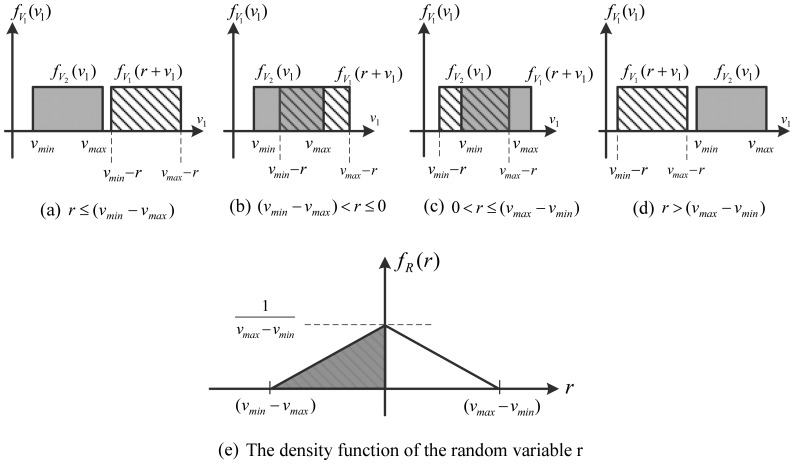
Derivation of the density function of the relative velocity *r*.

**Table 1.** Major activities during the sleep and active modes.

	**Major activities during sleep mode**			**Major activities during active mode**

	**Location-based**	**Time-based**	**Interrupt-driven**	
**Sensor**	Off	Off	Off	Busy sensing the pipeline environment (e.g., pressure sensing)
**RFID reader**	Communicating with RFID tags	Off	Off	Communicating with RFID tags
**Microcontroller**	Busy processing the RFID information for the purpose of localization and to make decision to wakeup	Busy running the timer and processing time information for the purpose of making decision to wakeup	Off	Busy doing two things: Collection and storage of sensor data Processing of RFID information for localization

**Table 2.** Design parameters.

**Parameter**	**Description**	**Unit**
*T*	Total node trip period	seconds
*T_A_*	The active period of a node	seconds
*T_rd (A)_*	The period of communication between an RFID reader and a tag	seconds
*N*	Number of deployed nodes	-
*E_n_*	Energy consumed by the *n*^th^ node	Joules
*M*	Total number of RFID tags	-
*m*	Number of RFID tags in a pipeline segment	-
*D*	Total pipeline distance	km
Δ*d*	Distance between RFID tags	meter
*f_s_*	Sensor sampling rate	Samples per second
*W_RFID_*, *W_sensor_*	Data width of the RFID and the sensor sample respectively	Bytes
*P_rd (idle)_*, *P_rd (A)_*	Power consumed by RFID reader in idle and active modes respectively	Watts
*P_C_*	Average power consumed by the microcontroller	Watts
*P_s_*	Power consumed by the sensor	Watts

**Table 3.** Analysis data (*D* = 400 km, *T_RD(A)_* = 2 s).

**Parameter**	**Min. Value**	**Max. Value**	**Increment Step**
*_T_*	10 h	70 h	20 h
*_N_*	1 node	60 nodes	5 nodes
Δ*d*	10 m	500 m	40 m to increment the minimum value and 50 m elsewhere

**Table 4.** Sensor node components and typical power consumed.

**Component**	**Brand**	**Power Specs.**
Pressure sensor	Intersema MS5541C [19]	18 μW
Microcontroller	LPC1102 Cortex-M0 [20]	16.5 mW
RFID reader	Tagsense ZR-232 Active Tag Reader [21]	9.9 μW (idle), 3.3 mW (communicating)
RFID tag	Tagsense ZT-50 Active RFID Tag [17]	9 μW (idle), 60 mW (communicating)

**Table 5.** Comparison of the energy results as obtained by simulation and the proposed equation.

	**Location-based Energy Results**				**Time-based Energy Results**				**Interrupt-Driven Energy Results**			

	**Simulation (in Joules)**	**Equation (in Joules)**	**Absolute Error (in Joules)**	**Relative Error (%)**	**Simulation (in Joules)**	**Equation (in Joules)**	**Absolute Error (in Joules)**	**Relative Error (%)**	**Simulation (in Joules)**	**Equation (in Joules)**	**Absolute Error (in Joules)**	**Relative Error (%)**
Max.	4429	4429	5.38	6.67	4429	4429	5.21	6.67	4429	4429	5.21	6.67
Min.	9.5	10	0	0	9.5	10	0	0	9.5	10	0	0
Mean	1247	1248	**0.96**	**0.17**	1228	1229	**0.95**	**0.17**	80.4	80.4	**0.47**	**0.82**
Std. Dev.	-	-	1.08	0.38	-	-	1.078	0.38	-	-	0.66	1.02

## Appendix

### Analytical Proof of Pipeline Coverage

In the location-based technique, missing any part of the pipeline is not possible because the duty handover is always triggered by reaching certain fixed predefined points along the pipeline. Whenever an active node reaches the next handover point it will stop immediately. At the same location, the next node to be in duty will wake up at that point as well. Thus, no matter which of the two nodes are faster than the other, handover will occur with guaranteed coverage for entire pipeline length.

In the interrupt-driven technique, duty handover is triggered by interrupting the sleeping node by the active node. Thus, the handover process implies dependency on the nodes and, hence, coverage is controlled and guaranteed.

For the time-based technique, handover is triggered by internal timers in the active and sleeping nodes. Assuming the timers are synchronized, the two nodes will handover duty at the same time instance. However, knowing that the two nodes can be in undetermined locations, it would be possible that the sleeping node (to be active) is ahead of the current active node. Obviously, the location gap between them is not monitored by either of them. A detailed analysis is presented here.

The randomness in the nodes location is due to the randomness in their forward velocity. Nodes, while travelling, are not heading perfectly forward, but there would be some slight drifting towards left or right. Thus, their motions have two components: *x*-component (moving forward) and *y*-component (moving right or left). It is the forward movement that counts in duty handover. Therefore, the forward velocity of a node is not fixed and neither is it deterministic. Thus, it has a random value *v* ranging between minimum velocity (*v_min_*) and maximum velocity (*v_max_*). For the sake of argument, that value is assumed to follow a uniform distribution within *v_min_* and *v_max_* of the density function *f_v_*(*v*)as shown in Figure A1.

The distance (*d*) that a node has travelled so far during time (*t*) can be considered as a random process while *v* is its random variable. The well-known velocity-time-distance equation can be used to describe that random process. That is:

(A1) d(v)=tv

Considering the two nodes involved in the duty handover, lets denote the active node distance as *d*_1_(*v*) while the sleeping node (to be active) as *d*_2_(*v*) If the forward velocities of the first node and the second node are *v*_1_ and *v*_2_ respectively, then the following expressions can be used:

(A2) $$d_{1}(v_{1})=tv_{1}$$

(A3) $$d_{2}(v_{2})=tv_{2}$$

The handover that occurs at instance *t* will not imply missing any part of the pipeline if and only if *d*_2_(*v*_2_) ≤ *d*_1_(*v*_1_)at that instant of time. That is:

(A4) $$d_{2}(v_{2})-d_{1}(v_{1})\leq 0$$

(A5) $$t(v_{2}-v_{1})\leq 0$$

(A6) tr≤0

where, *r*=(*v*_2_−*v*_1_) is the relative velocity between the two nodes. Note that *r* is yet a random variable of density function *f_R_*(*r*). Since the random variable *r* is the result of the difference between two random variables, *f_R_*(*r*) can be determined using the following relationship [22]:

(A7) $$f_{R}(r)=\int_{-\infty }^{\infty }f_{V_{1}}(r+v_{1})f_{V_{2}}(v_{1})\;dv_{1}$$

For *r* ≤ (*v_min_* − *v_max_*), the value of *f_R_*(*r*) is 0 since there will be no overlap between *f_v_*(*v*_1_).and *f_v_*(*v*_2_) (Figure A2a). Likewise, when *r* > (*v_max_* − *v_min_*), they do not overlap and, hence, the value of *f_R_*(*r*) is 0 (Figure A2d).

On the other hand, when (*v_max_* − *v_min_*) < *r* ≤ 0, the functions *f*_*V*_1__(*v*_1_) and *f*_*V*_2__(*v*_2_)overlap from *v_min_* − *r* to *v_max_* (Figure A2b). That is:

(A8) $$f_{R}(r)=\int_{v_{\text{min}}-r}^{v_{\text{max}}}\frac{1}{{\left(v_{\text{max}}-v_{\text{min}}\right)}^{2}}dv_{1}=\frac{v_{\text{max}}-v_{\text{min}}+r}{{\left(v_{\text{max}}-v_{\text{min}}\right)}^{2}}\;\text{for}\;v_{\text{min}}-v_{\text{max}}<r\leq 0$$

Similarly, when 0 < *r*≤ (*v_max_* − *v_min_*) the functions *f*_*V*_1__(*v*_1_) and *f*_*V*_2__(*v*_2_) overlap from *v_min_* to *v_max_* − *r* (Figure A2c). That is:

(A9) $$f_{R}(r)=\int_{v_{\text{min}}}^{v_{\text{max}}-r}\frac{1}{{\left(v_{\text{max}}-v_{\text{min}}\right)}^{2}}dv_{1}=\frac{v_{\text{max}}-v_{\text{min}}-r}{{\left(v_{\text{max}}-v_{\text{min}}\right)}^{2}}\;\text{for}\;0<r\leq v_{\text{max}}-v_{\text{min}}$$

In summary, *f_R_*(*r*) can be expressed by the following equation which is also depicted in Figure A2e:

(A10) $$f_{R}(r)=\{\begin{array}{cc} \frac{v_{\text{max}}-v_{\text{min}}+r}{{\left(v_{\text{max}}-v_{\text{min}}\right)}^{2}} & \text{for}\;v_{\text{min}}-v_{\text{max}}<r\leq 0\\ \frac{v_{\text{max}}-v_{\text{min}}-r}{{\left(v_{\text{max}}-v_{\text{min}}\right)}^{2}} & \text{for}\;0<r\leq v_{\text{max}}-v_{\text{min}}\\ 0 & \text{otherwise} \end{array}$$

The grey region in Figure A2e indicates the interval when *r* is negative which means that the sleeping node is slower than the active node. Hence, the area under the grey half of the curve is the probability that the handover process does not end up with missing parts of the pipeline unmonitored. That area is equal to 0.5. On the other hand, the remaining part of the curve is associated with positive values of *r* meaning that the sleeping node is faster than the active node. Therefore, when handing over duty to the sleeping node, it wakes up in a location that is ahead of the active node, which implies a gap between the two nodes that is not covered by them.

In conclusion, fifty percent of the handover processes are expected to end up with missing some parts of the pipeline. When deploying *N* nodes in a mission, there will be *N*–1 handovers throughout the pipeline length, fifty percent of which are expected to result in monitoring gaps. That is, (*N*–1)/2 gaps are expected. Note that, a gap length (like any other distance) is not a function of *N*, but it is a function of velocity and time. Therefore, deploying more nodes in the time-based technique implies increasing the occurrence of handovers and, hence, increasing the total number of potential gaps.
